# Influence of Frailty Syndrome on Outcomes of Cardiovascular Surgery in Elderly Patients

**DOI:** 10.21470/1678-9741-2024-0402

**Published:** 2025-10-27

**Authors:** Felipe Borsu de Salles, Gabriella Zanin Fighera, Veridiana Borges Costa, Kalil Hussein Khalil, Renato Abdala Karam Kalil, Bruna Eibel

**Affiliations:** 1 Instituto de Cardiologia, Porto Alegre, Rio Grande do Sul, Brazil; 2 Instituto do Coração do Hospital das Clínicas da Faculdade de Medicina da Universidade de São Paulo, São Paulo, São Paulo, Brazil; 3 Instituto de Cardiologia do Rio Grande do Sul - Fundação Universitária de Cardiologia, Porto Alegre, Rio Grande do Sul, Brazil

**Keywords:** Cardiac Surgery, Frailty, Mortality, Major Adverse Cardiovascular Cerebral Events.

## Abstract

**Introduction:**

Frailty syndrome is a significant risk factor for elderly patients undergoing cardiovascular surgery. However, there is no consensus on which criteria are most effective for assessing frailty in this context.

**Objective:**

This study aimed to evaluate the relationship between different widely cited frailty syndrome criteria and postoperative morbidity and mortality.

**Methods:**

Patients aged ≥ 60 years scheduled for coronary artery bypass graft, valve, and/or ascending aortic surgery were assessed for frailty preoperatively. Frailty was defined by Clinical Frailty Scale (CFS) ≥ 4, Katz Index ≥ 1, Short Physical Performance Battery (SPPB) ≤ 6, Fried Frailty Phenotype (FFP) ≥ 3 or abnormal values in 15-feet gait speed (GS) test, or hand grip strength. Clinical outcomes, including mortality and major adverse cardiovascular and cerebral events (MACCE), were assessed 30 days post-surgery.

**Results:**

Among 137 patients (70.1% male, mean age 69.43 ± 5.98 years), frailty prevalence ranged from 13.1% to 43.1%, depending on criterion, with no significant differences by age strata or surgery type. At 30-day follow-up, mortality was 5.1% (n = 7), and a total of 29 MACCE (21.1%) were recorded. Patients identified as frail by the FFP, CFS, SPPB, and GS criteria showed a significant association with mortality and MACCE. Multivariate analysis indicated FFP and CFS as independent risk factors for MACCE with equivalent prognostic prediction.

**Conclusion:**

Frailty is a prevalent condition among elderly patients undergoing cardiovascular surgery and is associated with mortality and morbidity. Frailty defined by FFP and CFS criteria was independently associated with higher MACCE rates.

## INTRODUCTION

**Table:** 

Abbreviations, Acronyms & Symbols				
AUC	= Area under the curve		KI	= Katz Index
BMI	= Body mass index		MACCE	= Major adverse cardiovascular and cerebral events
CABG	= Coronary artery bypass grafting		OR	= Odds ratio
CFS	= Clinical Frailty Scale		PCI	= Percutaneous coronary intervention
CI	= Confidence interval		ROC	= Receiver operating characteristic
COPD	= Chronic obstructive pulmonary disease		SD	= Standard deviation
FFP	= Fried Frailty Phenotype		SPPB	= Short Physical Performance Battery
GS	= Gait speed		STS	= Society of Thoracic Surgeons
HG	= Hand grip		TAVI	= Transcatheter aortic valve implantation

Population aging is accompanied by an increase in the number of elderly individuals undergoing cardiovascular surgery^[1]^. The prevalence of comorbidities increases in elderly patients, which may raise the risk related to cardiac surgery. The decision to indicate a surgical procedure requires a careful assessment of the risks and benefits probabilities for each patient. In geriatric population, traditional risk scores are less accurate^[2]^. Understanding the specific clinical conditions of the geriatric population plays an important role in this decision-making. The reduction in scores’ accuracy among elderly patients may be influenced by frailty syndrome, a factor not included in current scoring methodologies.

Frailty syndrome is defined as a decline in resilience to stressors, such as cardiovascular surgery^[3]^. Multiple studies have demonstrated an association between frailty and increased incidence of morbidity and mortality in the elderly undergoing cardiovascular procedures^[4^-^6]^. Although there are several methods to identify frailty, there is no consensus in the literature regarding the most accurate predictive criteria. A recent review identified 67 different frailty syndrome definitions^[7]^, some are based on symptoms, physical and/or cognitive tests, laboratory and imaging exams, and even a combination of these. Efforts to identify the most reliable frailty criterion has led to various combinations of variables to achieve better predictive results and thus the creation of new scores^[8^,^9]^. This variability in criteria contributes to heterogeneous samples across studies, complicating the comparison of results and leading to an unusual wide range of incidence, from 4.1%^[4]^ up to 49.7%^[6]^.

Frailty syndrome scores have different performance according to the type of surgery performed and patient features^[8^,^10]^. A significant proportion of scientific studies predominantly focus on patient cohorts from Europe and North America, with a greater emphasis on transcatheter interventions over sternotomy-based surgical procedures. Also, cultural and sociodemographic characteristics of the elderly population may influence the calibration and discrimination of these tests. Life expectancy is related to socioeconomic development, in addition to worse health indicators in the elderly^[11]^. All these differences across populations may influence the performance of frailty scores, so it is necessary to evaluate the predictive success of these tests in our population.

The aims of this study are to compare six widely cited definitions of frailty syndrome, to assess their respective associations with postoperative morbidity and mortality, and to determine the prevalence of frailty syndrome in this clinical setting according to each definition in a Brazilian cohort of elderly patients scheduled for conventional cardiac surgery.

## METHODS

### Study Design

This is a prospective cohort of consecutive elderly patients undergoing cardiovascular surgery selected at the Instituto de Cardiologia/Fundação Universitária de Cardiologia (Porto Alegre, Brazil) between July and December 2019. On the eve of scheduled cardiovascular surgeries, the researchers screened patients for study inclusion and applied a questionnaire and physical tests. The treating physicians were unaware of the results of these tests. The primary outcome was defined as a composite endpoint of mortality or postoperative major adverse cardiovascular and cerebral events (MACCE), assessed at 30 days postoperatively. This endpoint included death, acute myocardial infarction, stroke, non-fatal cardiac arrest, new-onset acute renal failure requiring dialysis, and hospital readmission. Data collection was conducted in accordance with the protocols established by the BYPASS Registry^[12]^.

### Patients

Inclusion criterion was age ≥ 60 years, based on Brazilian Federal Law^[13]^, which defines this age cutoff as elderly. All participants underwent scheduled standard sternotomy cardiac surgery, comprising coronary artery bypass grafting, valve replacement or repair, and/or ascending aortic surgery. Patients initially recruited but not submitted to surgery were excluded from the analysis (n = 1). Exclusion criteria were non-cardiac surgery associated and emergency surgery. Ethical approval was granted by the institutional review board (CAAE: 87473118.6.0000.5333), and participants provided written informed consent.

### Frailty Definitions

This study analyzed the following frailty scores: Fried Frailty Phenotype (FFP)^[3]^, Short Physical Performance Battery (SPPB)^[14]^, Rockwood Clinical Frailty Scale (CFS)^[15]^, and Katz Index (KI)^[16]^. Additionally, the 15-feet gait speed (GS) test and hand grip (HG) strength, components of the FFP, were also used as frailty criteria. GS and HG thresholds for frailty identification were adjusted for sex and body mass index (BMI), as defined by the FFP^[3]^. Participants with FFP scores ≥ 3, CFS class ≥ 4, KI scores ≥ 1, or SPPB scores ≤ 6 were classified as frail.

Frailty was assessed using six validated tools, each capturing distinct dimensions of the syndrome. FFP^[3]^ evaluates frailty based on five criteria:

1. Unintentional weight loss.
2. Self-reported exhaustion.
3. Low physical activity.
4. Slow GS.
5. Weak HG strength.

SPPB^[14]^ is a scale of 0 to 12. SPPB measures physical function through:

1. Balance test.
2. Chair stands test.
3. GS test.

The Rockwood CFS^[15]^ is a nine-point scale that categorizes frailty based on clinical judgment, ranging from "very fit" to "terminally ill". Similar to the New York Heart Association classification for heart failure symptoms, which uses a four-point scale to assess effort tolerance, the CFS evaluates autonomy and independence across a broader nine-point spectrum.

KI^[16]^ assesses independence in activities of daily living, including bathing, dressing, toileting, transferring, continence, and feeding. Additionally, the 15-feet GS test and HG, both components of the FFP, were used to objectively measure physical performance. HG strength was indexed by sex and BMI, while GS was indexed by sex and height. These adjustments ensure that the measurements account for physiological differences among individuals. Together, these tools provide a comprehensive evaluation of frailty across physical, functional, and clinical domains.

### Statistical Analysis

Quantitative variables were described in absolute values, measures of central tendency, and dispersion. For normally distributed data, the mean and standard deviation were used, while for non-normally distributed data, the median and interquartile range were reported. Categorical variables were presented by absolute and relative frequencies. Comparison of groups was assessed by Student’s *t*-test for normally distributed quantitative variables, by Mann-Whitney U test for quantitative variables not normally distributed, by analysis of variance for multiple groups analysis, and by Chi-square test for categorical variables. For low frequencies, Fisher’s exact test was used. Multivariable analysis was performed for identification of independent risk factors for MACCE and receiver operating characteristic (ROC) area under the curve (AUC) with its respective confidence limits for risk prediction. All tests were performed with IBM SPSS Statistics for Windows, version 20.0 (IBM Corp., Armonk, NY, USA), and we used a two-tailed alpha significance level of 0.05.

### Sample Size Calculation

A sample size calculation indicated that 136 participants would be required to detect a two-fold increase in mortality and MACCE between frail and fit patients, with a two-tailed α of 0.05 and a β of 0.20 (80% power), based on prior mortality data^[4]^.

## RESULTS

**Fig. 1:**
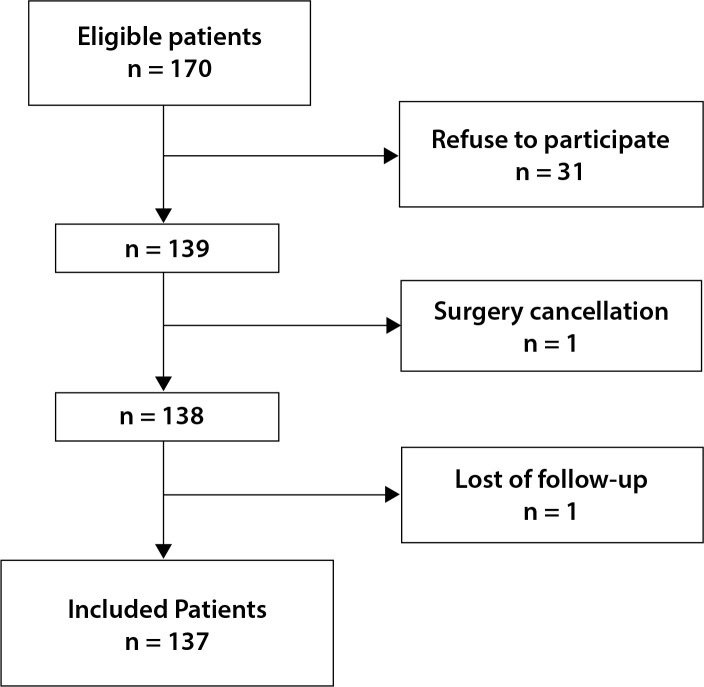
Flowchart of sample selection.

Of the 170 patients screened, 31 declined participation, one was excluded due to surgery cancellation, and one was lost to follow-up (Figure 1). Consequently, 137 patients were included in the analysis, 70.1% (n = 96) were male, and the mean age was 69.43 ± 5.98 years. Table 1 presents the characteristics of the study sample.

**Table 1:** Preoperative characteristics of the study sample.

	Total (n = 137)
Age, years, mean ± SD	69.43 ± 5.9
Male sex, % (n)	70.1% (96)
Diabetes mellitus, % (n)	34.3% (47)
Dyslipidemia, % (n)	37.2% (51)
Systemic arterial hypertension, % (n)	75.9% (104)
Smoking, % (n)	8% (11)
Ex-smoking, % (n)	43.8% (60)
Coronary artery disease, % (n)	56.9% (78)
Acute myocardial infarction, % (n)	29.9% (41)
Previous PCI, % (n)	21.2% (29)
Previous cardiovascular surgery, % (n)	2.9% (4)
Congestive heart failure, % (n)	37.2% (51)
Stroke, % (n)	8% (11)
Peripheral artery disease, % (n)	2.2% (3)
Chronic kidney failure, % (n)	4.4% (6)
COPD, % (n)	5.1% (7)
Active endocarditis, % (n)	2.2% (3)
Creatinine, mg/dl, mean ± SD	1.01 ± 0.30
Left ventricular ejection fraction, %, mean ± SD	58.8 ± 13
Hemoglobin, g/dl, mean ± SD	13.35 ± 1.6
Glycated hemoglobin, %, mean ± SD	6.54 ± 1.57
STS Score, %, mean ± SD	1.38% ± 1.04

COPD=chronic obstructive pulmonary disease; PCI=percutaneous coronary intervention; SD=standard deviation; STS=Society of Thoracic Surgeons

**Fig. 2:**
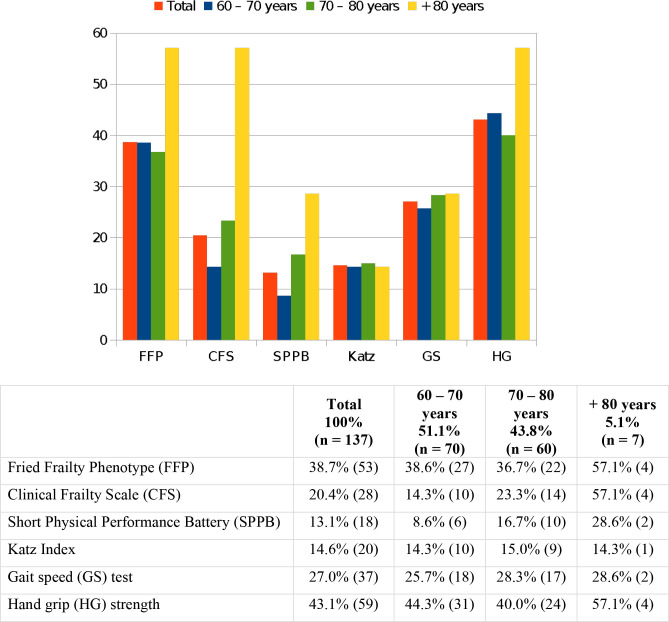
Prevalence of frailty according to different age stratum (%).

The prevalence of frailty ranged from 13.1% to 43.1% depending on the criterion used. Frailty prevalence stratified by age (Table 2 and Figure 2) and type of surgery (Table 3) is presented. During the 30-day follow-up, mortality was 5.1% (n = 7), myocardium infarction occurred in eight patients (5.8%), stroke in six patients (4.4%), acute kidney injury in five patients (3.7%), and rehospitalization in three patients (2.2%), with a total of 29 MACCE events (21.1%).

**Table 2:** Prevalence of frailty and surgical risk scores according to different age stratum.

	Total	60 - 70 years	70 - 80 years	+ 80 years	*P*-value
	100%	51.1%	43.8%	5.1%	
	(n = 137)	(n = 70)	(n = 60)	(n = 7)	
Fried Frailty Phenotype	38.7% (53)	38.6% (27)	36.7% (22)	57.1% (4)	0.59
Clinical Frailty Scale	20.4% (28)	14.3% (10)	23.3% (14)	57.1% (4)	0.02^*^
Short Physical Performance Battery	13.1% (18)	8.6% (6)	16.7% (10)	28.6% (2)	0.14
Katz Index	14.6% (20)	14.3% (10)	15.0% (9)	14.3% (1)	1.00
Gait speed test	27.0% (37)	25.7% (18)	28.3% (17)	28.6% (2)	0.95
Hand grip strength	43.1% (59)	44.3% (31)	40.0% (24)	57.1% (4)	0.66
STS score	1.38 ± 1.04	1.08 ± 0.84	1.64 ± 1.19	2.08 ± 0.49	0.001^*^

* *P* < 0.05

STS=Society of Thoracic Surgeons

**Table 3:** Prevalence of frailty and surgical risk scores according to type of surgery.

	CABG	Valve	CABG + valve	Aorta	*P*-value
	62.0%	23.4%	10.2%	4.4%	
	(n = 85)	(n = 32)	(n = 14)	(n = 6)	
Fried Frailty Phenotype	35.3% (30)	46.9% (15)	50.0% (7)	16.7% (1)	0.38
Clinical Frailty Scale	18.8% (16)	21.9% (7)	28.6% (4)	16.7% (1)	0.85
Short Physical Performance Battery	11.8 % (10)	12.5% (4)	28.6% (4)	0% (0)	0.29
Katz Index	10.6% (9)	25.0% (8)	14.3% (2)	16.7% (1)	0.22
Gait speed test	25.9% (22)	28.1% (9)	42.9% (6)	0% (0)	0.28
Hand grip strength	43.5% (37)	43.8% (14)	42.9% (6)	33.3% (2)	0.99
STS score	1.11 ± 0.97	1.82 ± 1.24	2.07 ± 0.40	1.18 ± 0.19	< 0.001^*^

* *P* < 0.05

CABG=coronary artery bypass grafting; STS=Society of Thoracic Surgeons

**Fig. 3:**
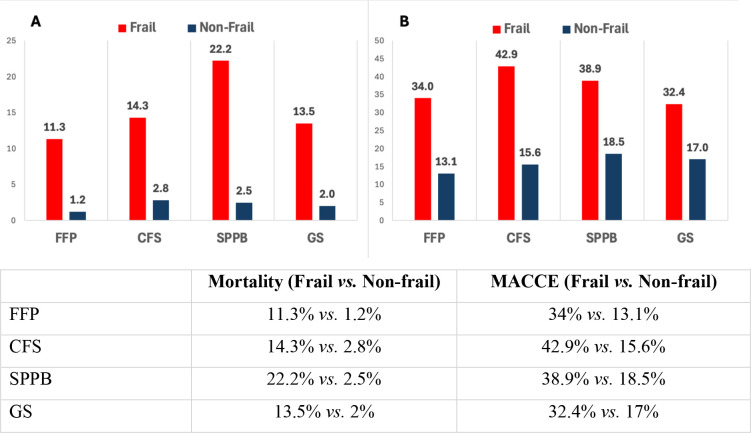
A) Mortality according to different frailty scores (%). B) Morbidity according to different frailty scores (%). CFS=Clinical Frailty Scale; FFP=Fried Frailty Phenotype; GS=Gait speed; MACCE=major adverse cardiovascular and cerebral events; SPPB=Short Physical Performance Battery.

**Fig. 4:**
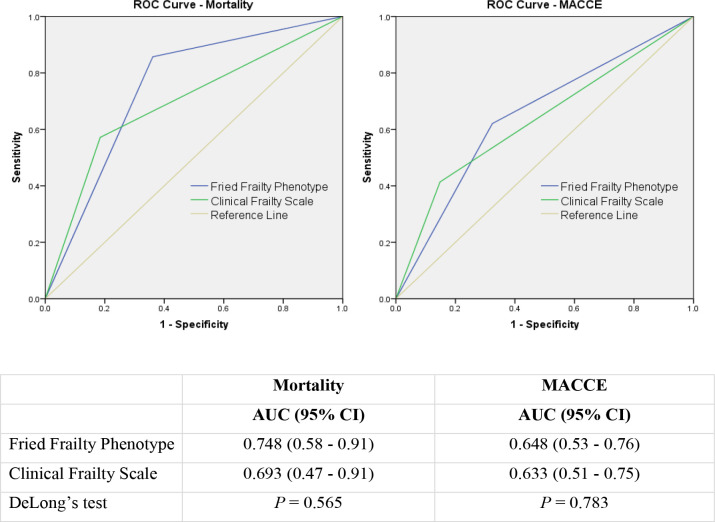
A) ROC curve for mortality. B) ROC curve for morbidity. AUC=area under the curve; CI=confidence interval; MACCE=major adverse cardiovascular and cerebral events; ROC=receiver operating characteristic.

Patients defined as frail by the FFP, Rockwood CFS, SPPB, and GS test had association with negative clinical outcomes in univariate analysis. These findings and the respective odds ratio are described in Table 4 and Figure 3. KI and HG were not related to death or MACCE (not described). A multivariate logistic regression analysis, adjusted for the Society of Thoracic Surgeons (STS) Score, indicates FFP and CFS as independent risk factors for MACCE (Table 5). Multivariate logistic regression was performed only for MACCE and not for mortality because there were not enough events (n = 7) to perform a useful analysis. The area under the ROC curve was used as a measure of performance (Figure 4), demonstrating better predictive probability for FFP than CFS for mortality (AUC 0.749 *vs.* 0.693) and MACCE (AUC 0.648 *vs.* 0.633). DeLong’s test demonstrated no significant difference in predictive value between the assessed models, with *P*-values of 0.565 for mortality and 0.783 for MACCE.

**Table 4:** Frailty scores associated with mortality and morbidity.

	Frail	Fit	OR (95% CI)	*P*-value
Fried Frailty Phenotype	38.7% (53)	61.3% (84)		
Mortality	11.3% (6)	1.2% (1)	10.56 (1.24 - 90.69)	0.014^*^
MACCE	34.0% (18)	13.1% (11)	3.41 (1.46 - 8.00)	0.005^*^
Clinical Frailty Scale	20.4% (28)	79.6% (109)		
Mortality	14.3% (4)	2.8% (3)	5.89 (1.24 - 28.06)	0.032^*^
MACCE	42.9% (12)	15.6% (17)	4.06 (1.63 - 10.08)	0.003^*^
Short Physical Performance Battery	13.1% (18)	86.9% (119)		
Mortality	22.2% (4)	2.5% (3)	11.05 (2.24 - 54.52)	0.006^*^
MACCE	38.9% (7)	18.5% (22)	2.80 (0.98 - 8.06)	0.063
Gait speed test	27.0% (37)	73.0% (100)		
Mortality	13.5% (5)	2% (2)	7.66 (1.42 - 41.40)	0.016^*^
MACCE	32.4% (12)	17% (17)	2.34 (0.99 - 5.56)	0.061
Hand grip	43% (59)	56.9% (78)		
Mortality	6.8% (4)	3.8% (3)	1.81 (0.39 - 8.45)	0.44
MACCE	22% (13)	20.5% (16)	1.09 (0.48 - 2.5)	0.82
Katz Index	14.6% (20)	85.4% (117)		
Mortality	10% (2)	4.3% (5)	2.49 (0.45 - 13.8)	0.28
MACCE	25% (5)	20.5% (24)	1.29 (0.43 - 3.9)	0.65

* *P* < 0.05; univariate logistic regression

CI=confidence interval; MACCE=major adverse cardiovascular and cerebral events; OR=odds ratio

**Table 5:** Frailty scores associated with major adverse cardiovascular and cerebral events adjusted for the Society of Thoracic Surgeons Score.

	OR (95% CI)	*P*-value
Fried Frailty Phenotype	6.11 (1.26 - 7.48)	0.013 ^*^
Clinical Frailty Scale	6.74 (1.37 - 9.72)	0.009 ^*^

* *P* < 0.05; multivariate logistic regression

CI=confidence interval; OR=odds ratio

## DISCUSSION

Frailty is a prevalent condition among elderly patients undergoing cardiovascular surgery. Although a relatively recent risk factor, frailty is an important area of study. This paper presents data from a specific region, Porto Alegre, in the southern Brazilian context, which is characterized by one of Brazil’s highest sociodemographic indices, life expectancy, and healthy life expectancy^[11]^.

The study’s inclusion criterion of age 60 years aligns with Brazilian Federal Law^[13]^, which defines this cutoff as the threshold for senior status. While this threshold may impact the prevalence of frailty and its association with morbidity and mortality, it adheres to the Brazilian government’s definition of elderly and therefore is relevant to the local context. Brazilian law also designates a second cutoff of 80+ years as “priority seniors”, but this stratification was not performed in the current study due to small sample in this stratum. Frailty syndrome studies commonly focus on older populations, reflecting the established understanding that frail individuals are generally older than their non-frail counterparts^[4]^. Two southeastern Brazilian cohort studies have explored the influence of frailty in cardiovascular surgery: one used the same cutoff point^[17]^, while the other selected older patients (*i.e.* ≥ 65 years)^[18]^. Another Brazilian population-based cohort study analyzed the prevalence of frailty in adults over 18 years old^[19]^. This study demonstrated that frailty is present even in younger cardiovascular patients; however, its prevalence is significantly higher in older age groups.

This study observed a wide range of frailty prevalence, varying based on the criteria used, consistent with previous research. A prior Brazilian cohort, utilizing the CFS, reported a 65.1% frailty prevalence^[18]^, while a study using GS found 42.3% of participants to be frail. In both cohorts, the prevalence was higher than observed in our sample. Our frailty prevalence ranged from 13.1% using the SPPB to 43.1% using HG. This prevalence was independent of surgery type or age subgroups. Only the CFS showed an increase in frailty prevalence in the older stratum, with other frailty assessment tools yielding no similar findings. This lack of difference may be attributed to smaller group size following stratification. As our study was designed to evaluate clinical outcomes rather than frailty prevalence, further studies with larger samples or focused on prevalence within specific age groups or surgery types are needed to clarify potential relationships.

Various frailty assessments were associated with postoperative mortality and the incidence of MACCE. However, neither the HG nor KI independently identified patients at greater risk of death or MACCE. In the first study evaluating frailty in cardiovascular surgery context, KI demonstrated an association with hospital mortality^[4]^. In contrast to our study, that study’s sample was larger, older, and exhibited a lower prevalence of frailty. Others specific local characteristic of patient population may also contribute to the absence of an association between the KI and clinical outcomes. HG assessment provides information on a limited group of muscles and may not accurately reflect the overall musculoskeletal status of elderly patients. While HG identified the higher prevalence of frailty, it was not associated with mortality nor MACCE in our study. A recent meta-analysis of pooled data from patients with clinical cardiovascular disorders identified HG as an independent predictor of mortality and hospital admission^[20]^. These patients were managed clinically, without undergoing cardiac surgery, which may explain the observed discrepancy. Despite the established utility of HG in the rehabilitation setting^[21]^, there is insufficient evidence to support its use as a prognostic tool in cardiac surgery.

In univariate analysis, mortality was associated with FFP, CFS, SPPB, and GS. FFP and CFS also had a significant relationship with MACCE, while GS and SPPB showed a nominal increase in MACCE but with a borderline non-statistical difference. After risk adjustment with the STS Score in a multivariate logistic regression, FFP and CFS remained good predictors of MACCE. Risk adjustment was not performed for mortality due to the reduced number of events (n = 7), which limits this statistical analysis.

Frailty syndrome alone has demonstrated the ability to predict morbidity and mortality, with accuracy comparable to, or even exceeding, that of traditional risk scores^[8^,^10]^. Afilalo et al.^[8]^ identified GS as a superior prognostic frailty test as compared to the FFP and others frailty and disability scales. This finding led to the inclusion of GS in the STS database and a cohort of 15,171 patients^[5]^, demonstrating a gradual increase in mortality with decreasing walking speed. Consistent with these studies, our research also found GS to be associated with mortality in our sample; however, only FFP and CFS showed associations with both mortality and MACCE. A meta-analysis of 66,448 patients who underwent cardiac surgery^[22]^ revealed that frailty and pre-frailty were associated with a two-fold and 1.5-fold increase in adjusted operative mortality, respectively, as well as increased in adjusted perioperative complications. Furthermore, frailty was associated with an approximately five-fold increased risk of non-home discharge. Frailty is prevalent and associated with higher mortality even in non-elderly patients undergoing cardiac surgery^[23]^.

Frailty syndrome has been tested as a prognostic tool, primarily in North America and Europe. Studies on the association of frailty syndrome in elderly people undergoing cardiovascular surgery are lacking in developing countries. Similar to our study, two previous Brazilian cohorts indicated frailty as a risk factor for adverse outcomes. Salles et al.^[17]^ showed that frailty defined by GS could improve European System for Cardiac Operative Risk Evaluation II prediction and is an independent risk factor for MACCE. Rodrigues et al.^[18]^ used the CFS as frailty criterion and demonstrated longer mechanical ventilation time, longer intensive care unit stay, longer hospitalization, increased MACCE, and higher mortality. In both cohorts, only one criterion for frailty was used, thus preventing a comparison of different definitions to determine which is most suitable for the local population. In our study, both the FFP and the CFS showed comparable AUC values in predicting mortality and MACCE. Given the comparable AUC values and the fact that CFS is easy to use (requiring no additional devices, unlike the FFP's need for a HG dynamometer), the CFS emerges as a potentially preferred tool for assessing frailty syndrome.

Although the criteria for defining frailty vary across studies, frailty is consistently identified as a significant risk factor in both general cardiac surgery^[24]^ and specific procedures such as aortic valve replacement^[9^,^25]^. This prognostic value extends to less invasive interventions, including off-pump coronary artery bypass grafting^[26]^ or minimally invasive coronary artery bypass grafting surgery^[27]^. Further research is warranted to determine the most effective tool for each surgical type.

The observed mortality rates and MACCE in this study were consistent with previously published findings^[12]^. Elderly patients commonly present with comorbidities that impact postoperative outcomes, as evidenced by the significant increase in STS Score among older patients in our study. An increase in mortality and morbidity within the older stratum was anticipated; however, this analysis was not conducted due to the limited sample following stratification.

Frailty is associated with increased mortality following standard sternotomy cardiovascular surgery and plays a particularly significant role in transcatheter aortic valve implantation (TAVI). Frailty holds an important prognostic value for morbidity, hospital stay, and mortality, even in minimally invasive cardiac procedures such as TAVI^[28]^, and has been investigated as a justification for utilizing less invasive approaches. Given that our study exclusively included patients undergoing traditional sternotomy, the findings are not comparable to those from TAVI or other minimally invasive surgical techniques.

### Limitations

Limitations of this study include its single-center data, predominantly scheduled surgeries, exclusively standard sternotomy surgery, and multiple types of surgery, which may not be representative of other hospitals and series. Inclusion criteria cutoff of 60 years included younger patients than other publications but follows elderly definitions according to Brazilian Federal Law.

Patients' refusal to enroll (31 of 170 eligible patients, 18.2%) in this study may be related to frailty or fear of any physical test, insecurity due to unfamiliarity with the test administrator, coupled with a fear of triggering symptoms and thus may influence final results. The reason for refusal was not documented. Adopting frailty criteria based solely on anamnesis, without physical testing, such as CFS, may reduce study participation refusal rates.

The small number of mortality events (n = 7) resulted in a very wide confidence interval for the univariate analysis of mortality and precluded the possibility of performing a multivariate analysis. Another limitation of this study is the lack of analysis of social or demographic characteristics, which may differ across regions and could potentially influence clinical outcomes.

Frailty was evaluated in patients the night before their scheduled procedure. While preoperative optimization was not performed within this study, our findings indicate a significant association between frailty and increased MACCE. Consequently, we propose that the identification of frailty through preoperative screening may enable clinicians to implement specific interventions aimed at optimizing patient status and reducing the risk of postoperative complications.

## CONCLUSION

Frailty is a prevalent condition among elderly patients undergoing cardiovascular surgery, and its association with higher mortality and MACCE may vary depending on the diagnostic criterion used. HG strength testing and KI are established frailty assessment tools, but their applicability and predictive capacity in the specific context of elderly patients scheduled for elective cardiovascular surgery requires further evaluation. Knowing the risk profile of frail patients will be possible to reinforce preoperative strategies and appropriate care during hospital stay. Based on these data, risk-benefit estimation of cardiac surgery should include frailty evaluation to identify higher risk patients. The FFP and the Rockwood CFS were independently sensitive as predictors for MACCE in elderly submitted to cardiac surgery.

The Rockwood CFS demonstrated predictive performance comparable to that of the FFP. However, the CFS is easier to learn and apply and does not require additional devices. Therefore, we recommend adopting the CFS as the standard tool for frailty assessment.

## Data Availability

The authors declare that the data will be available upon request to the authors.

## Artificial Intelligence Usage

The authors declare use of free version of ChatGPT 3.0 for orthography correction for English version of this text.
